# A Case of Endometrial Tissue Containing Meckel's Diverticulum in an Adult

**DOI:** 10.7759/cureus.44843

**Published:** 2023-09-07

**Authors:** Brandol Wolfenbarger, Kevin M Cottingham

**Affiliations:** 1 General Surgery, Alabama College of Osteopathic Medicine, Dothan, USA; 2 General Surgery, Cullman Regional Medical Center, Cullman, USA

**Keywords:** endometriosis, ectopic endometrial tissue, chronic abdominal pain, anatomical pathology, gastrointestinal obstruction, meckel's diverticulum in adults

## Abstract

A Meckel's diverticulum is one of the most common congenital causes of small bowel obstruction. The lack of a more common gastric-containing tissue and older age of symptom onset can lead to difficulties with preoperative diagnosis. This case demonstrates an adult with chronic abdominal pain with recurrent small bowel obstruction that was found to be a rare ectopic endometrial tissue containing a Meckel's diverticulum following a diagnostic laparotomy. The barriers to diagnostics and factors affecting the age of onset are discussed with a focus on demonstrating the importance of surgical intervention for small bowel obstruction due to a Meckel's diverticulum and on the significance of a rare histological finding.

## Introduction

A Meckel's diverticulum (also known as Meckel diverticulum or congenital ileal diverticulum) is a congenital malformation resulting from the failed obliteration of the proximal portion of the omphalomesenteric (vitelline) duct [1]. Usually disappearing by gestational week nine, the persistence of this structure can lead to several anomalies with a Meckel's diverticulum being the most common [1]. The resulting 3 to 6 cm outpouching arises from the antimesenteric border of the ileum and is located within 75 cm of the ileocecal junction in 75% of cases [1]. Although usually an incidental finding, it can lead to complications such as intestinal obstruction or inflammation of the diverticulum resulting in bleeding, ulceration, or perforation [2]. Retrospective studies have found that more than half of symptomatic patients were younger than 10 years old; however, complications can occur at any age [3]. As few as 5.7% of symptomatic Meckel's diverticula are diagnosed preoperatively [4]. This case report highlights the challenges of preoperative diagnosis and the role of diagnostic surgery in Meckel’s diverticula. In this case, a 43-year-old female presented with a multi-year history of chronic abdominal pain and recurrent small bowel obstruction. Her chronic pain was misdiagnosed but eventually discovered to be a Meckel's diverticulum containing ectopic endometrial tissue.

## Case presentation

A 43-year-old female presented to the emergency department with a 24-hour history of severe upper abdominal pain. The pain was gradual in onset, worsened by food, and associated with nausea and vomiting. The patient denied any alleviating factors, constipation, diarrhea, urinary abnormalities, chest pain, or back pain. She had a history of multiple similar episodes of small bowel obstructions, which resolved without surgical intervention. Despite negative biopsy results, her previous doctor encouraged her to undergo treatment for Crohn’s disease. Her symptoms prompted an evaluation for hereditary angioedema, but the workup was negative. Her past medical history included hypothyroidism and occasional heart palpitations. Her surgical history was significant for a cholecystectomy, C-section, and hysterectomy. Her physical exam revealed a soft abdomen with normal bowel sounds and moderate tenderness in the epigastric and periumbilical areas with no palpable mass.

Laboratory tests were significant for an elevated white blood cell count of 17.72 10^3^/uL and a normal lipase of 99 U/L. An abdominal CT scan without contrast demonstrated distention of the stomach and dilated loops of the small bowel without any clear source of obstruction.

**Figure 1:**
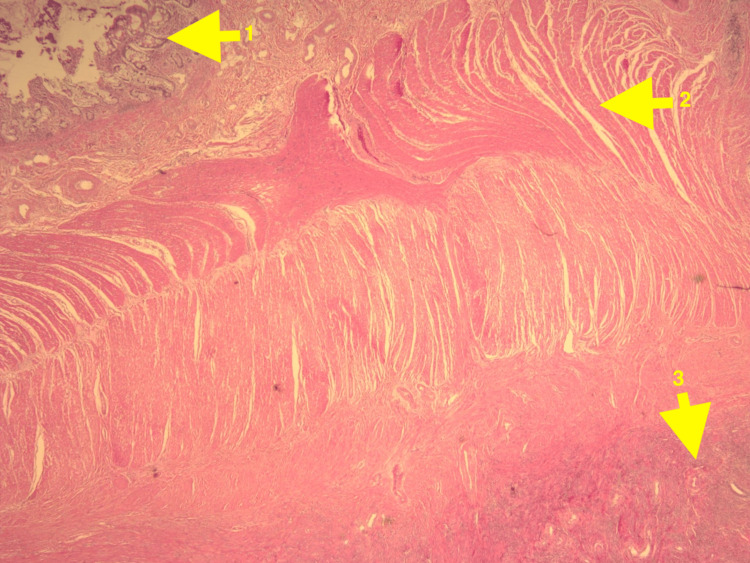
Section of Meckel's diverticulum and adjacent bowel on scanning power (4x). 1. Area of ileal tissue 2. Area of smooth muscle 3. Several endometrial glands and stromal tissue within the wall of the diverticulum.

**Figure 2:**
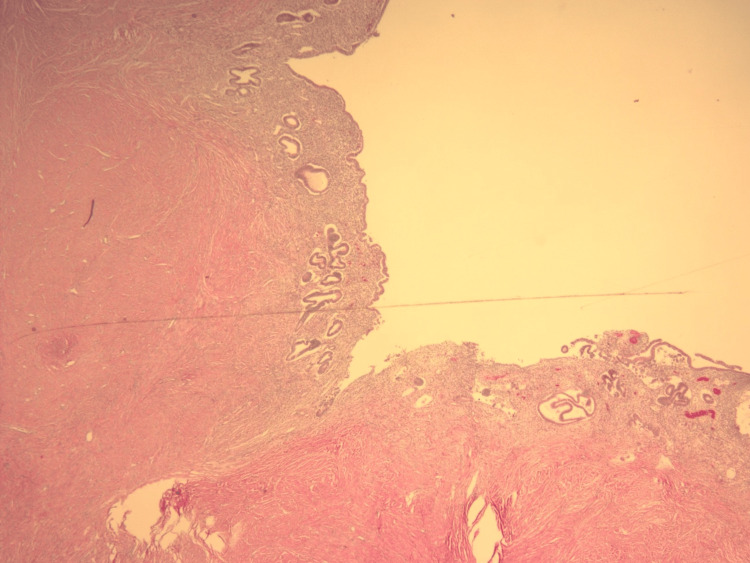
Section of Meckel's diverticulum on scanning power (4x) showing heterotopic endometrial tissue.

**Figure 3:**
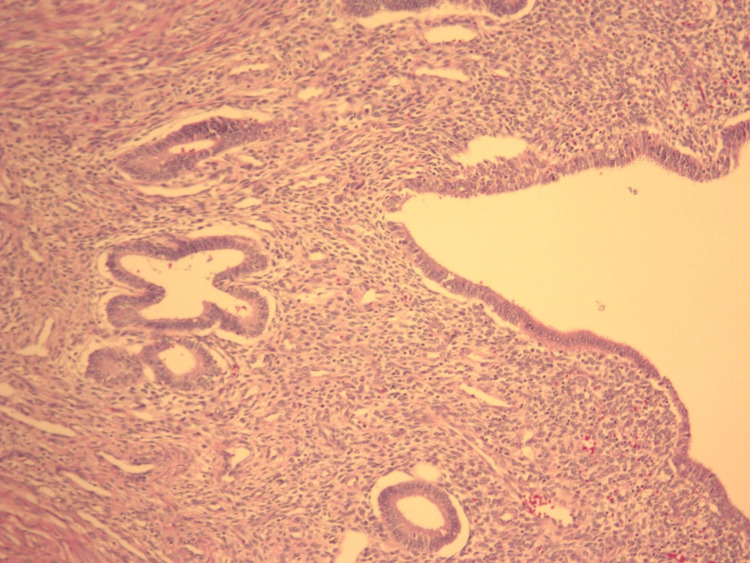
Section of Meckel's diverticulum on low power (10x) showing heterotopic endometrial tissue.

The patient was initially treated conservatively symptomatically with adequate hydration and analgesia with bowel decompression. Due to a history of numerous admissions for bowel rest and IV fluids for symptomatic relief, the decision was made to explore surgically. The expected outcome was the common finding of adhesions which would simply require enterolysis. The actual outcome was encountering an inflamed Meckel's ileal diverticulum along with associated inflammatory adhesions that required enterolysis and small bowel resection. Laparoscopic exploration revealed a location of adhesions approximately 60 cm proximal to the ileocecal valve. Extensive enterolysis identified a 3 x 3 x 2.6 cm diverticulum with dilation of the small bowel proximal to this point due to obstruction. No other lesions were identified. The patient had an uncomplicated postoperative recovery, with complete resolution of bowel obstruction, and was discharged one day after surgery. Histologic examination of hematoxylin and eosin-stained sections of the resected bowel revealed that the diverticulum contained endometrial tissue and benign small bowel tissue. The remainder of the resected bowel contained benign small bowel tissue. Due to the adherence of the adjacent small bowel with the diverticulum, a thickened area of smooth muscle can be seen separating normal ileal tissue and heterotopic endometrial tissue in Figure 1. The luminal surface of the diverticulum was lined by ectopic endometrial glands and endometrial stroma, as shown in Figure 2 and Figure 3. 

## Discussion

In this case report, we present a 43-year-old female with chronic abdominal pain and recurrent small bowel obstruction due to an etiology less frequently observed in adult populations. In addition, our case revealed ectopic endometrial tissue involving a Meckel’s diverticulum, a finding that is scarcely documented in the literature.

The most common clinical presentation of a Meckel's diverticulum in adults is bleeding from ectopic gastric mucosa, while in children it is obstruction due to intussusception [5]. The reported prevalence of Meckel's diverticula in the general population ranges from 0.3% to 2.9% based on eight studies [3]. Zani et al. performed a systematic review analyzing a total of 31,499 patients in the form of autopsy studies which found a prevalence of 1.2% [6]. Hansen et al. found a wide range of reported incidences of symptomatic Meckel's diverticula ranging from 9.0% to 71.1% [3]. Zani et al. determined a lifetime incidence of symptomatic Meckel's diverticula of 4.2% based on the aforementioned autopsies they reviewed and reported hospital admissions [6]. The largest non-age discriminatory patient population studied was by Park et al. which found an incidence of 16.1% in a retrospective study of 1,476 patients [3,5]. Retrospective studies and database queries have found that symptomatic Meckel's diverticula have a male predominance with a male-to-female ratio ranging from 1.5:1 to 4:1 [3]. The most significant factor for developing a symptomatic Meckel's diverticulum is the presence of ectopic tissue lining the walls of the diverticulum. Gastric tissue is the most common ectopic tissue and is present in 4.6% to 71% of symptomatic patients. Ectopic pancreatic tissue is second to that at 0% to 12%. Contrast these to non-symptomatic Meckel's diverticula with 0% to 18.2% containing gastric tissue and 0% to 5% containing pancreatic tissue [3]. These data are summarized in Table 1. In both symptomatic and non-symptomatic cases, less common ectopic tissues can be seen, as demonstrated by this case report. After an extensive literature review, we identified only two instances of similar ectopic endometrial tissue involving Meckel’s diverticulum. Interestingly, both were classified as endometriosis [7,8]. Endometriosis describes the presence of functional endometrial tissue outside the uterus and is often accompanied by hormone-responsive chronic abdominal pain and menstrual irregularities [9]. Several theories have been proposed for the etiology of endometriosis [9]. Sampson’s theory of retrograde menstruation suggests endometrial tissue seeding via fallopian tubes during menstruation [9,10]. Minh’s theory proposes transformation of peritoneal mesothelium [9,11]. Lymphatic and blood system spread, as well as pro-angiogenic factors like vascular endothelial growth factor, may also play a role [9]. In both of these cases, the ectopic tissue was confined to the diverticulum, and in one case, it was identified as the source of small bowel obstruction [8]. Won simply noted the unique finding as an extension of regional enteritis without attributing it to a contributory role [7]. The frequency of gastrointestinal tract involvement varies by source however endometriosis of the gastrointestinal tract overall is common with rectosigmoid making up the majority of the locations (72%) [12]. Of note, this patient had previously undergone a hysterectomy for undisclosed reasons; however, it is unknown if there is a potential relationship. No other lesions were identified during the procedure, and the patient's medical history did not include endometriosis. Whether this nomenclature is the most appropriate for this specific entity is difficult to discern given its rarity. However, by definition, the case we present and those previously reported can be classified as endometriosis.

**Table 1:** Presentation and frequency of ectopic tissue presence in symptomatic and asymptomatic Meckel's diverticulum. Source: [3]

Presentation	Gastric Tissue Present	Pancreatic Tissue Present
Symptomatic Meckel's Diverticulum	4.6% to 71%	0% to 12%
Asymptomatic Meckel's Diverticulum	0% to 12%	0% to 5%

With most cases being asymptomatic, Meckel's diverticula are often found incidentally during laparoscopy or laparotomy when looking for the source of GI bleeding or obstruction [3]. Standard imaging modalities such as ultrasound, X-ray, angiography, CT, and MRI can identify a blind-ended pouch coming off of the ileum. However, the sensitivity and specificity of these tests are low [3]. For preoperative localization, the technetium 99m pertechnetate scan is the most common test for detecting the more common gastric tissue-containing Meckel's diverticulum, with some limitations. For children, it is effective with a sensitivity of 80-90% and specificity of 95%. However, in adults, the test is less useful, with a sensitivity of 62% and specificity of 9% [13]. Larger ectopic gastric tissue is more easily detected but also more likely to hemorrhage at a young age, leading to adults being more likely to have smaller areas of ectopic gastric tissue [13]. Furthermore, if the diverticulum does not contain ectopic gastric tissue or is positioned behind normally gastric tissue-containing organs [3], the scan is unlikely to identify the diverticulum. Treatment for a symptomatic Meckel's diverticulum is surgical resection [3]. Postoperative morbidity was 5.3%, commonly due to wound infection and postoperative ileus [3]. Complications of resection have been shown to be similar between symptomatic and non-symptomatic Meckel's diverticula [3]. In one study, the complication rate of a non-symptomatic Meckel's diverticulum was lower than a symptomatic one [3]. Benefits and risks of prophylactic diverticulectomy of incidental cases have been addressed with conflicting results [3,5]. When an incidental Meckel's diverticulum is detected in imaging studies, retrospective reviews do not recommend elective resection [5,6,14-17]. However, in the case of an incidental diverticulum discovered during abdominal exploration, surgeons should evaluate each patient's lifetime risk of developing complications related to the diverticulum [5,15,18]. Table 2 shows an approach proposed by Robijn et al. that offers a risk score to guide resection decisions [18].

**Table 2:** A risk score for guiding resection decisions of incidentally detected Meckel's diverticula. Source: [18]

Risk Factor		Points
Sex	Male	3
	Female	1
Age	<45 years	2
	>= 45 years	1
Length of diverticulum	>2 cm	2
	<= 2 cm	1
Presence of fibrous band	Yes	3
	No	0
Risk score	Total points	
Resection recommended for scores >= 6		

There are limited data regarding Meckel's diverticulum-associated tumors; however, malignant transformation seems to be uncommon, with reported incidences of 0.5% to 3.2% [4,15,17,19]. The majority of reported tumors are benign but malignant types have also been reported [15,20,21].

## Conclusions

A Meckel's diverticulum, despite being a common differential for small bowel obstruction in children, is a rare cause of small bowel obstruction in adults. This case describes recurrent small bowel obstructions secondary to a Meckel's diverticulum that had a rare histopathological finding of ectopic endometrial tissue. Difficulties in diagnosing a Meckel's diverticulum in non-traditional patient populations can lead to unnecessary recurrence and worsening of patient symptoms. Increasing awareness of Meckel's diverticulum as a potential cause of small bowel obstruction in adults and recognizing the limitations of preoperative diagnostics can decrease the time to appropriate treatment.
